# Clinical adoption of virtual reality in mental health is challenged by lack of high-quality research

**DOI:** 10.1038/s44184-024-00069-8

**Published:** 2024-05-16

**Authors:** Benjamin Selaskowski, Annika Wiebe, Kyra Kannen, Laura Asché, Julian Pakos, Alexandra Philipsen, Niclas Braun

**Affiliations:** Department of Psychiatry and Psychotherapy, Faculty of Medicine, University of Bonn, Bonn, Germany

**Keywords:** Diagnosis, Therapeutics, Translational research, Psychiatric disorders

## Abstract

Virtual reality has been found effective for some mental disorders, while for many others weak methodology prevents conclusive evidence. Similar to other digital technologies, the field has particular demands for conducting clinical research which currently remain poorly addressed. In this commentary, we discuss the unique issues associated with the incorporation of virtual reality in clinical research. In addition, we elaborate on the possibility that these challenges may also be consequences of current funding and publication schemes, and speculate on specific improvement approaches that might be more compatible with the characteristics of clinical virtual reality research.

Virtual reality (VR) is an innovative and advanced technology for research to generate and control computer-simulated, realistic, and interactive environments into which participants can be immersed. In comparison to conventional technologies, the most relevant benefits of VR for clinical use are the ability to create immersion and a sense of presence (for details, see Table 1) while keeping the patient in a controlled and safe environment that still offers scope for individualization. Aside from its use in surgery and rehabilitation, clinical VR appears to have the greatest potential in mental health^1^. The ability to collect real-time data during participants’ performance in these controlled virtual environments is of special interest in this field, as affected individuals tend to exhibit specific behaviors or experience symptoms in certain contexts that are difficult to simulate by other means. In particular, the enhanced ecological validity associated with VR (i.e., generalizability to the real world) has attracted considerable attention.

## Current state

**Fig. 1:**
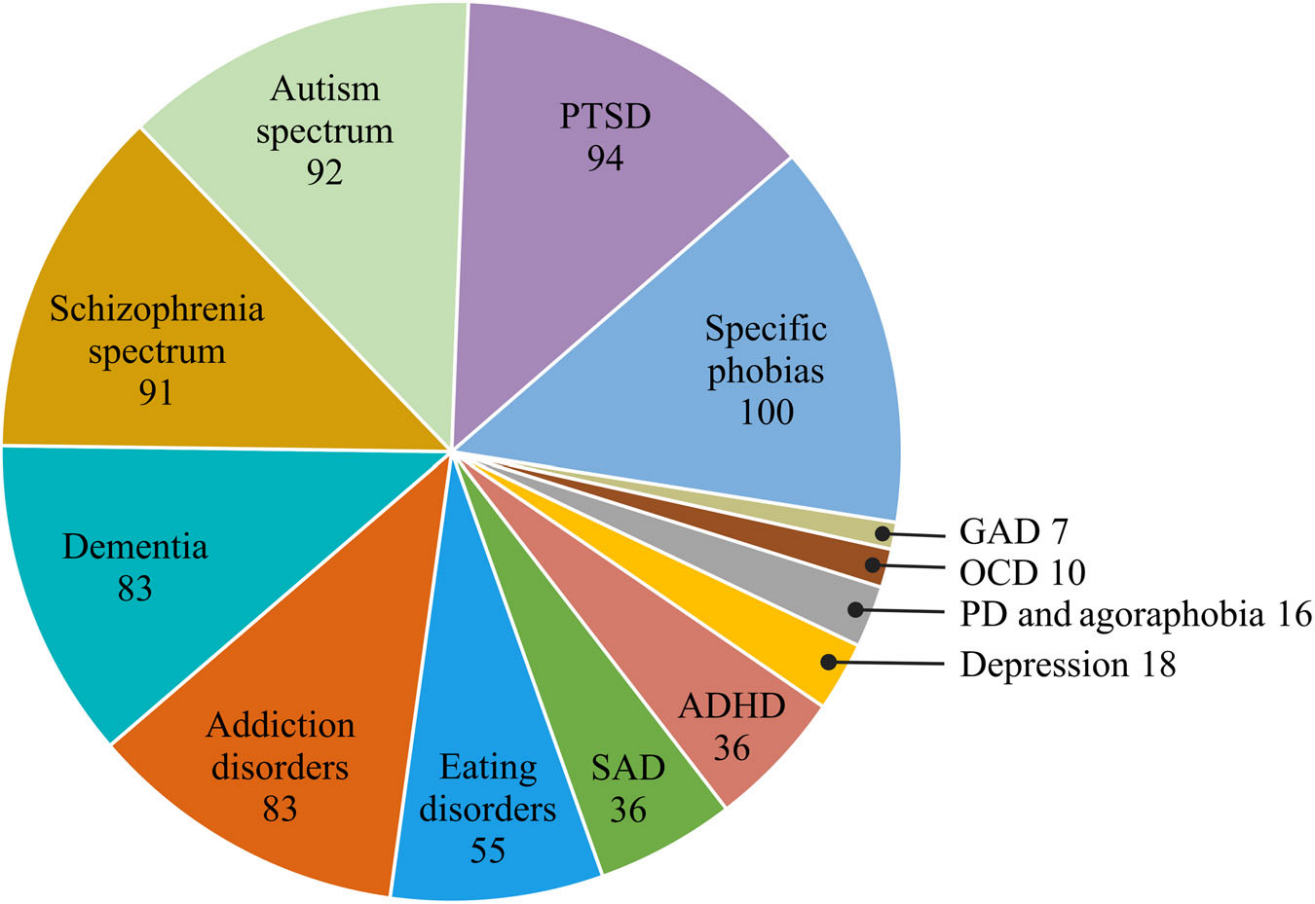
Proportion of clinical virtual reality studies published in mental health until May 2022 per disorder, including assessment and treatment approaches^2^. ADHD attention-deficit/hyperactivity disorder, GAD general anxiety disorder, OCD obsessive-compulsive disorder, SAD social anxiety disorder, PD panic disorder, PTSD posttraumatic stress disorder.

Comprising 721 studies, our research group recently published the largest systematic review on the use of VR in mental health to date, suggesting that VR will most likely find its way into routine psychiatric care within several domains^2^. Although the number of studies is still small for some disorders, particularly generalized anxiety disorder, panic disorder and agoraphobia, obsessive-compulsive disorder, and depression, a substantial number of studies have already been published for other disorders such as specific phobias, autism spectrum disorder, posttraumatic stress disorder, dementia, schizophrenia spectrum disorders, and addiction disorders (see Fig. 1). VR has shown promise in both improving the accuracy of assessments, such as in ADHD, and enhancing treatment effects, for instance, via virtual (cue) exposure therapy in anxiety, phobias, post-traumatic stress disorder, and addiction^2^. However, consistent with previous research^3,4^, we have also emphasized that the lack of rigorous scientific investigation has prevented conclusive evidence for many mental disorders. The current quality of clinical VR research has even been described as the “Wild West,” with a focus on technique rather than theoretical rationale, primarily descriptive evaluation, insufficient power, and many retrospective analyses^5^. In the following, we aim to compare specific study quality characteristics of VR studies with those of non-VR studies in mental health and discuss potential factors underlying the lack of larger-scale implementation of rigorous trial design to date.

**Fig. 2:**
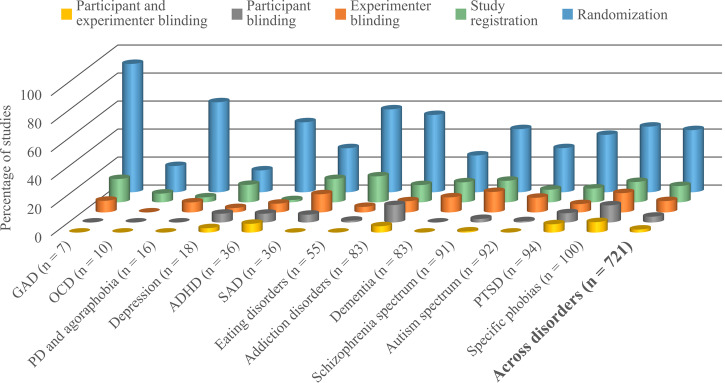
Disorder-specific proportion of studies that used the following study techniques to minimize potential bias: blinding of participants and experimenters (yellow), blinding of participants only (gray) or experimenters only (orange), study registration (green), and randomization (blue). Mean scores across disorders were 2.2%, 4.2%, 8.1%, 11.7%, and 44.4%, respectively. Total numbers of included studies for each disorder are presented in parentheses. ADHD attention-deficit/hyperactivity disorder, GAD general anxiety disorder, OCD obsessive-compulsive disorder, SAD social anxiety disorder, PD panic disorder, PTSD posttraumatic stress disorder.

The quality of a study is usually assessed based on a set of characteristics that may introduce bias (i.e., systematic error) to an outcome measurement, such as the type of control condition, participant allocation procedures, study registration, and concealment of group allocation. Although likely biased toward higher quality since all included studies were registered, a review of 3258 trials across mental health found randomization in 86.4% and blinding in 67.2% (45.6% double-blind) of studies^6^. In contrast, in mental health VR research (see Fig. 2), we observed randomization in only 44.4% of the studies and blinding of participants or experimenters in only 10.1% (2.2% double-blind). Moreover, risk of bias composite scores for VR studies grouped by disorder consistently yielded mean ratings greater than 50% (range, 0–100%), indicating significant methodological limitations^2^. Another concern in this research field is small sample size^7,8^, which significantly reduces the reliability of the evidence and probability of successful replication. Previous reviews of studies in clinical psychology^9^ and mental health^10^ have reported median sample sizes of 90 and 61, respectively, clearly exceeding the median sample size of 36 identified for VR mental health studies^2^. These “astonishing” research gaps in scientific robustness, as referred to earlier^4^, seem to persist and continue to preclude firm conclusions on many aspects.

## Challenges

The reasons for the deficiencies in study quality may be multifaceted. The interdisciplinarity emphasized in clinical VR research appears to complicate study conduction, as experts from both computer science and clinical research are often required. For example, technical experts might design high-level scenarios but rely less on sound theoretical rationales than clinical mental health experts. Given that standardized, validated, and open-access VR applications are widely lacking^11^, clinical VR projects often involve an effortful, time-consuming two-step process: the development of a VR application and subsequent validation in a clinical trial. As a result of often tightly calculated project timelines, researchers are then forced to compromise between development and trial periods. Consequently, clinical evaluations of immature applications or small exploratory clinical studies of professionally developed applications that barely meet the minimum requirements for publication in clinical journals frequently emerge. The current focus of the development of VR applications in mental health is therefore predominantly centered on preliminary phases of clinical evaluation. While these early evaluations provide a means of assessing potential benefits and risks (primarily Phase 1 and Phase 2 trials in clinical treatment evaluation), conclusive evidence only emerges from subsequent large-scale, mostly multicenter studies (Phase 3 and Phase 4 trials). These trials ultimately allow for more robust conclusions to be drawn with regard to generalizability and meaningful comparisons to existing standard treatments. However, these large-scale studies incur high costs, which in the field of VR are further exacerbated by the costs of technical implementation and the small number of existing publicly available, standardized scenarios. Recently, initial approaches have evolved that aim to develop easy-to-use, low-effort platforms that allow for the generation of VR applications without in-depth programming knowledge^12^. However, the extent to which inherent challenges, such as trade-offs between customizability and simplicity, can be addressed to enable clinical researchers from different fields and with a variety of demands to implement VR applications more efficiently is yet to be determined. Given the pace at which the field of VR is evolving, and in light of non-validated clinical VR applications being prematurely made available to the public^13^, the need for high-quality research to identify what makes VR effective and, more importantly, when VR is not appropriate for clinical service is growing^14^.

## Ethical considerations

Accordingly, the current state of evidence also does not contribute to resolving ongoing ethical debates regarding various issues related to VR use in patients with mental disorders^15^. For instance, disorder-specific characteristics such as social isolation in depression could be of concern for participation in certain VR experiments. Considering a group therapy program in which patients meet virtually to promote social interaction, does transition to real-world social contact need to be imposed at some point? Or is it acceptable to comply with patients’ preferences to remain in an online environment, as this might be perceived as less distressing? The unclear implications of virtual contact for social isolation may therefore raise ethical concerns, especially given that vulnerable patients themselves may be more inclined to choose the “seductive” VR technology without fully anticipating consequences^16^. These may be serious and, given the association with social withdrawal, could range from suicidal thoughts to attempts^17^.

Likewise, infrequent and unstandardized reporting of side effects in mental health VR research causes barriers to the interpretation of clinical suitability^18^. Corresponding to other instruments evaluated for clinical use, a standardized framework for assessing adverse events is urgently required. In this context, not only cybersickness (i.e., a type of motion sickness caused by sensory mismatch between experienced visual and other sensory cues in virtual reality environments; for a review, see^19^), photosensitivity and photosensitive seizures (i.e., exposure to certain patterns, color changes, or light frequencies that provoke seizures; for a review, see^20^) but especially potential accompanying psychological symptoms, such as depersonalization or derealization, and their course under repeated exposure should be systematically assessed.

Ultimately, rigorous scientific evidence can guide emerging ethical considerations and must be the foundation for transitioning new technologies into clinical use. For instance, the proposal of therapeutic alternativism states that, for vulnerable patients, genuine human contact is likely preferable to human-machine interaction^16^. Although seemingly plausible, the extent to which this conclusion is not the result of a bias toward the familiar must be objectively examined based on evidence. Recent publication of studies in the field of automated VR may provide initial evidence in this regard. For instance, the rigorously conducted THRIVE study^21^, a four-session automated cognitive VR intervention for patients with persistent persecutory delusions, showed basic feasibility and effective symptom reduction, but no superiority over VR mental relaxation therapy. Although clinical staff was present during implementation, patient interaction was guided by a virtual coach. While no direct conclusions can be drawn about the efficacy of automated treatments, as time effects were not evaluated in an independent group, patient acceptance and safety were demonstrated. Future increasingly automated implementations would certainly have advantages in cost efficiency and treatment accessibility, but also carry risks of reduced human interaction that need to be investigated. For this reason, future clinical VR studies would certainly benefit from increased patient and public involvement (PPI) in addition to clinical and technical experts^22^.

## System adaptations

Overall, the current treatment evaluation system, including funding and publication frameworks, does not appear to adequately support the robust assessment of clinical efficacy of novel digital technologies. Particularly high costs arising from Phase 3 and Phase 4 studies in general, and those arising specifically from preceding technical development and implementation in this particular field, seem to have an impact on initiation of large-scale trials. Key elements of many funding instruments, such as preference for innovation, tend to promote the short-term creation of new VR applications rather than the improvement and robust validation of existing ones. Funding for confirmatory trials, on the other hand, often requires access to an existing, at least preliminarily feasible methodology, which in turn is an issue in the VR field given the limited number of standardized and openly available applications. These financial demands accumulate as individual research groups attempt implementation and often culminate in results of limited methodological value. A key component, especially in clinical VR, is therefore multicenter collaboration, even at smaller national scale. This not only enables the joint use of resources from different research groups with varying interdisciplinary expertise but provides the associated side effect of larger, more efficient sample size generation. Multicenter approaches will also directly address discussed methodological weaknesses, such as enhanced reproducibility and generalizability. Riva et al.^23^, for example, recently successfully completed one of the few clinical VR multicenter studies to date on their Secret Garden paradigm.

**Fig. 3:**
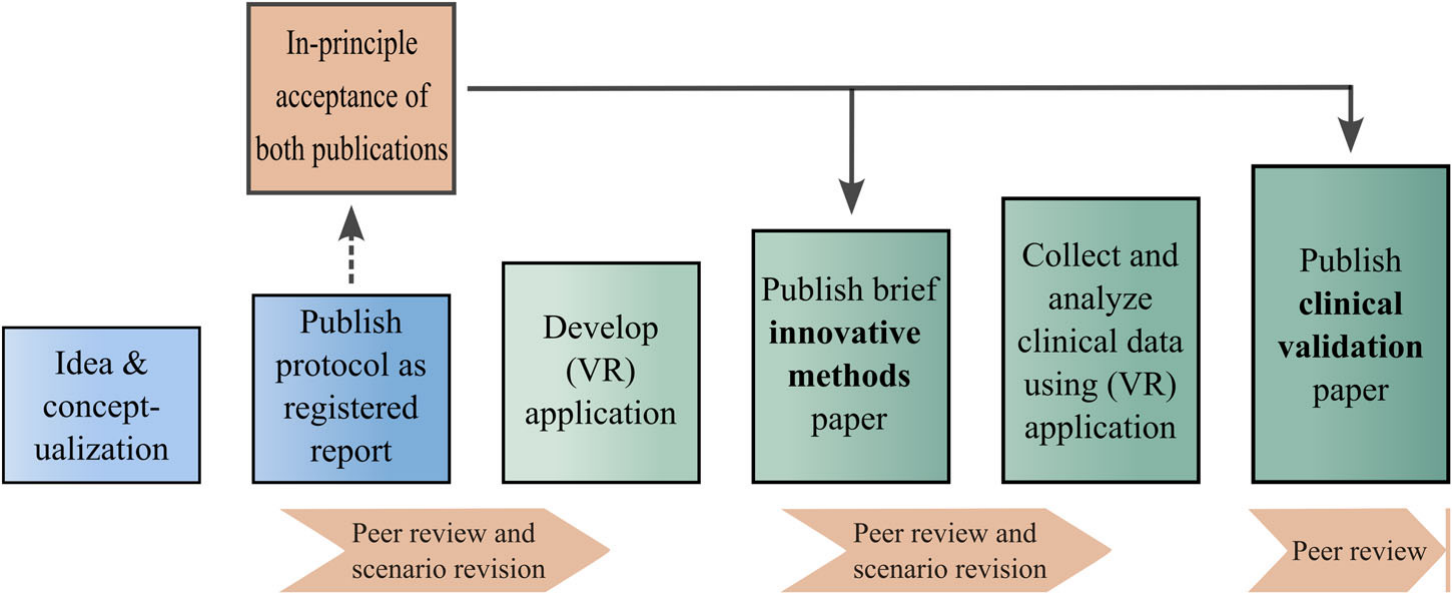
Accepted protocol-based dual publication model for the evaluation of digital technologies for clinical application. Initially, the study idea and concept will be peer-reviewed and publicly released. After in-principle acceptance, an innovative methods paper containing details of the developed (VR) application is published. Based on second stage peer-review, the (VR) application is revised and tested in a clinical trial, resulting in a second publication.

With respect to publishing practices, the lack of adaptability to the use of modern technologies is exemplified by manuscript requirements of many clinical journals, whose length constraints do not realistically allow for sufficiently detailed presentation of extensive methods such as VR. This practice applies not only to VR, but also to clinically required software and other digital technologies, further contributing to the lack of standardization in the field^24^. Therefore, we propose an accepted protocol-based dual publication model for the evaluation of digital technologies for clinical application (see Fig. 3) that builds on and extends current procedures for registered reports (i.e., peer-review of protocol and decision to publish before study begin). Herein, a study protocol is submitted that includes detailed descriptions of (1) the development of the VR application and (2) the subsequent clinical validation study. After completed peer review and revision of the protocol regarding VR application and study methodology, the publisher not only releases the protocol to the public (e.g., in dedicated journals or sections) but also provides in-principle acceptance for a two-fold publication process. Acceptance is maintained through compliance to the protocol and alignment with requested peer-review editing. Subsequently, once the application has been developed, a brief “innovative methods” paper will be published (ideally including PPI data). Thereafter, the original data from the conducted clinical trial will be published in a separate article. This early iterative review process of theoretical rationale, practical implementation, and study methodology could significantly enhance previous shortcomings by crediting VR researchers for well-considered design of VR applications as well as rigorous clinical validation. Once in-principle acceptance would qualify for inclusion as a funding criterion, there is a direct link between methodological quality assurance and adequate funding with synergy effects for all parties involved. Results of a transparent peer review process as the basis for a journal’s in-principle acceptance can be included in the funding proposal evaluation, and the preparation of the protocol for submission to the journal likewise provides the foundation for the funding proposal. The proposed model promotes several aspects of robust and transparent research that are of high value for funding agencies, such as certainty of result publication, methodological rigor, and decreased research practices related to publication bias.

## Outlook

Inconsistent evidence tends to magnify negative attitude toward a field. Although the initial hype around clinical VR seems to be subsiding with an increasing number of inconclusive scientific publications, there remains much potential to be explored. Capabilities that should be leveraged in the future include reducing waiting times for treatments through enhanced automated use without requirement of specialists; improving access to care through its scalability as a smartphone application (while patients without smartphone access could be served via low-cost rental devices by their respective healthcare provider); utilizing cost-effectiveness such as for use in developing countries; advancing personalized medicine, for example, by combining scenario functionalities to account for comorbidity; simultaneously measuring physiological responses (e.g., EEG and eye tracking); reducing reliance on retrospective self-reports to assess symptoms; and causing limited side effects, with specific features against VR-related cybersickness already developed^25^.

Until these aspects can be fully exploited, however, greater standardization is needed. While no reporting guidelines for clinical VR trials exist so far^26^, a consensus model for study conduction has been published^5^. Yet, while it appears that too many clinical VR studies may at most partially adopt this extensive framework, there are a number of easy-to-implement, basic steps for clinical trials that have a profound impact on study quality, as early as for Phase 1 and Phase 2 investigations (see Table 1).

**Table 1:** Exemplary, easy-to-implement quality indicators of clinical studies

Method	Description and examples	Achievement
A priori sample size determination	Ensuring sufficient power to detect meaningful effects	Reduce risk of Type II error, cost-efficiency
Preregistration of research plan	For example, preventing HARKing (i.e., forming hypotheses after the results are already known)	Increase reproducibility of results
Evidence informed outcome measure selection	For example, measures of immersion (i.e., the objective perception of being part of the virtual world based on sensory input) or presence (i.e., the subjective perception of being involved in the simulation). Benchmarks for immersion and standardized measurement tools for presence provide quantifiable parameters that allow analysis of its treatment impact.	Lack of immersion can lead to disruption of presence. In therapeutic applications, degree of presence can influence therapy effectiveness^28^. Optimization achievable via technical (e.g., improving haptic feedback), content (e.g., compelling narratives), and realistic interaction capabilities.
	For example, patient-reported outcome measures (PROMs) to capture patients’ perspectives on outcomes of healthcare interventions	Patient-centered approach, complementing clinical outcomes
Adequate control conditions	For example, distinguish effects of the clinical VR application itself from the VR medium^29^	Increase robustness of conclusions
Randomization	Gold standard for participant allocation	Prevent selection bias
Transparent reporting	For example, protocol noncompliance, or failure to maintain blinding	Improve objective interpretation and reproducibility of findings
Open-access scenario provision	Make the application available to other researchers in a form that allows for re-use	Future collaborations, external replication studies, transparency

Creating a research environment that places greater emphasis on both development and clinical validation, for example, based on the model proposed here, could greatly improve the quality of clinical VR research and subsequently have a significant impact on the mental health field. The increasing spread of VR software in clinical applications has also resulted in growing discussion about regulatory measures. Further specifications for the clinical use of VR systems are expected to be defined both in Europe as part of the Medical Device Regulation (EU MDR) and in the USA by the FDA, which will then first have to be fulfilled by economic operators. For clinical research, this traditionally tends to complicate project implementation until relevant cost-intensive classification procedures are completed (for a detailed discussion, see^27^). However, attempting to predict the future of clinical VR, the question likely is not when VR will be used routinely in clinical practice, but to what extent its use will be evidence-based.
